# Surgical and anesthetic management of paraganglioma diagnosed in a 2nd trimester parturient: Case report

**DOI:** 10.1016/j.amsu.2021.103094

**Published:** 2021-11-22

**Authors:** F. Aftiss, S. El Mezzeoui, G. El Aidouni, M. Merbouh, S. Nasri, R. Jabi, H. Bkiyar, I. Skikar, M. Bouziane, B. Housni

**Affiliations:** a Intensive Care Unit, Mohammed VI University Hospital Center, Oujda, Morocco; b Department of General Surgery, Mohamed VI University Hospital Center, Oujda, Morocco; c Department of Radiology, Mohamed VI University Hospital Center, Oujda, Morocco; d Faculty of Medicine and Pharmacy, Oujda, Morocco; e Oujda Medical Simulation Training Center, Morocco

**Keywords:** Paraganglioma, Pregnant women, Case report

## Abstract

**Introduction:**

Neuroendocrine tumors represent a rare entity whose diagnosis is based on clinical, biological and radiological arguments. When they are secreting, they expose the patient to serious complications that can be much more severe during pregnancy and engage the vital prognosis of both the mother and the fetus, which requires multidisciplinary management: anesthesiologist resuscitator - obstetrician – endocrinologist.

**Case presentation:**

In our article, we report the case of a patient with an estimated pregnancy at 25 weeks of amenorrhea (WA) with a history of 3 miscarriages related to atypical gravid hypertension.

The treatment consisted of preoperative medical preparation followed by removal of the paraganglioma and postoperative monitoring. The maternal-fetal evolution was favorable.

**Conclusion:**

The non-negligible morbi-mortality of this type of tumors require a multidisciplinary management.

## Highlights

• Neuroendocrine tumors represent a rare entity whose diagnosis is based on clinical, biological and radiological arguments.
• The treatment consisted of preoperative medical preparation, removal of the paraganglioma and postoperative monitoring.
• The anesthetic and surgical management of pheochromocytomas and paragangliomas in pregnant women presents a real challenge.

## 1 Introduction

Pheochromocytomas and paragangliomas are rare tumors that are considered a subset of a group of tumors called neuroendocrine tumors originating from adrenomedullary chromaffin cells that typically produce one or more catecholamines [1].

The diagnosis is first suspected clinically and then confirmed based on a combination of clinical, biological and radiological arguments. They are generally manifested by a paroxysmal hypertension often resistant to medical treatment with a triad: headache, sweat, palpitation otherwise there are atypical forms which should not be ignored [2,3].

The association with pregnancy is even rarer (incidence 0.002–0.007%). These tumors have a serious impact if not diagnosed and managed properly [4]. Therefore, these tumors should be investigated in the presence of any hypertension in a young and pregnant woman.

In our article we report the case of a pregnant patient with an estimated pregnancy of 25WA + 5 days who presented 3 miscarriages secondary to gravidic hypertension on an unrecognized paraganglioma. We will also discuss the anesthetic and the perioperative management of the paraganglioma surgical removal under laparoscopy.

## 2 Case presentation

A pregnant female, 34 years old, fourth gestation primiparity with current pregnancy estimated at 25 weeks of amenorrhea (WA) + 5 days, diabetic for 1 year, initially on oral antidiabetic drugs and then insulin therapy, hypertensive for 2 years on alpha methyldopa with a history of 3 miscarriages secondary to hypertensive peaks.

During the 4th pregnancy, the patient was hospitalized at 20 WA in the endocrinology department for an etiological assessment of a hypertensive crisis at 180/110 mmHg associated with headaches, palpitations and hot flashes, without proteinuria or edema.

In front of these symptoms the diagnosis of a secretory neuroendocrine tumor is suspected, and a biological assessment including urinary and plasma catecholamines (metanephrine and normetanephrine) came back positive after eliminating other causes of secondary hypertension (nephropathy, renal artery stenosis or hyperaldosteronism), by renal evaluation, renal artery Doppler ultrasound and renin-angiotensin-aldosterone system exploration.

Urinary dosages showed Metanephrines at 0.87 μmol/24 h (normal: 0.20–1), high Normetanephrine 24.14 μmol/24 h (normal: 0.4–2.10), While plasma dosages showed a Metanephrine level of 0.10 nmol/l (normal: < 0.33 nmol/l), a high level of Normetanephrine of 14.02 nmol (normal: <1.07 nmol/l). A thyroid workup was performed to rule out multiple endocrine neoplasia (MEN) returning normal: TSH 1.821 (normal: 0.340–5.330), anti-thyroperoxidase Ac < 0.8 IU/ml (normal <0.8).

**Fig. 1:**
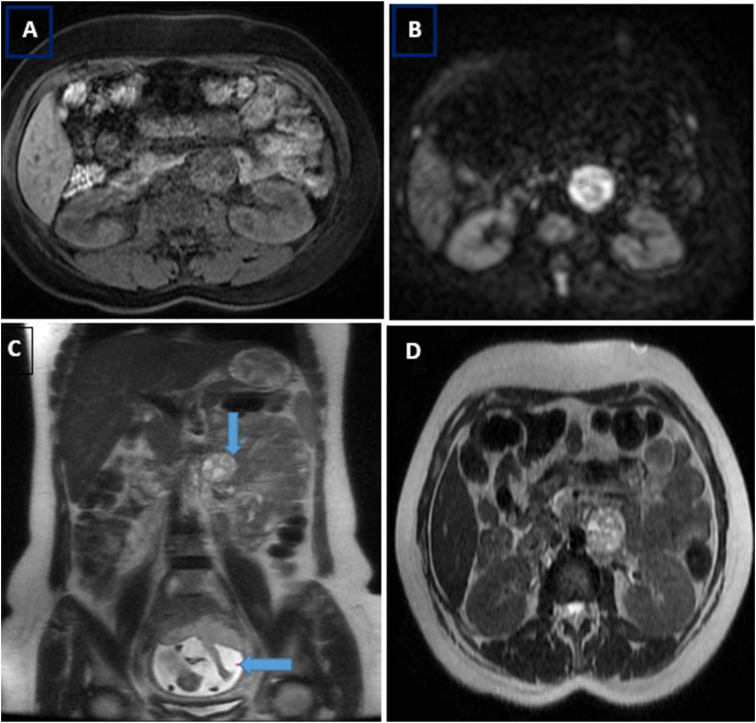
Abdominal MRI in sequences: Axial T1 (A), axial diffusion b1000 (B), coronal (C) and axial T2 (D) showing a round mass, well-limited, retroperitoneal left lateroaortic in isosignal T1, hypersignal T2 and diffusion, containing areas of fluid signal measuring 36 mm in diameter. Note: a gravid uterus.

Abdominal-pelvic magnetic resonance imaging (MRI) showing an abdominal latero-aortic mass measuring 36 * 33 mm, corresponding to paraganglioma without any other obvious location [Fig. 1].

For evaluation of maternal and fetal impact of the tumor:

Clinical examination including cardiovascular examination was normal. With an ECG that did not show rhythm or repolarization disorders or left ventricular hypertrophy.

A standard biological workup (blood cell count, platelet count, hemostasis, blood ionogram, blood sugar-HBA1c, renal and hepatic function) was unremarkable.

On the obstetrical level, the clinical examination and the obstetrical ultrasound showed a monofetal pregnancy with no uterine or fetal abnormalities.

After placing the patient on medical treatment and scheduling her for surgery, she was declared discharged with an ambulatory follow-up. At 25 weeks of amenorrhea; the patient was admitted to the operating room for laparoscopic removal of her paraganglioma.

In the operating room, considering the risk of perioperative morbidity related to severe per and postoperative hemodynamic instability (hypertensive crisis with sometimes rhythm disorder, coronary ischemia, left ventricular failure and hypotension secondary to a sudden release of catecholamines: to stress, painful stimulation due to intubation and incision, insufflation and manipulation of the tumor, abrupt and profound hypotension by sudden decrease of catecholamines at the fall of the part and venous clamping) and in front of the obligation to maintain a uterine perfusion and a good oxygenation of the fetus while avoiding maternal hypoxemia, the anesthetic management was as follows:

1. Monitoring:

Hemodynamic: heart rate (HR), blood pressure (BP)

Respiratory: respiratory rate, pulse oxygenometry (SpO2), capnography.

ECG and ST segment monitoring.

Cardiac output monitoring by pulse wave contour analysis.

2. Left jugular venous line (for central venous pressure monitoring and vasoactive drugs perfusion)
3. A right femoral arterial line with invasive arterial pressure monitoring
4. Heating of the patient to prevent hypothermia

After installation of the patient in left lateral decubitus proclive 30°, a pre-oxygenation was started, and an anesthetic induction was done by: 150mg Propofol 150μg Fentanyl, 50 mg Atracurium 50mg; intubation by tube No. 6.5 cm; maintenance of anesthesia was provided by: Propofol IVOC system with concentration of: 2ug/ml.

Maintenance of etCO2 32–35 mmHG with insufflation pressures not exceeding 12 mmHG (8–10 mmHG) and close monitoring of blood glucose due to the risk of blood glucose imbalance.

During tumor manipulation 3 episodes of arterial hypertension peaks were observed) (180/110–195/120-200/130 mmHg controlled by reinforcement of analgesia by reinjections of fentanyl 50 μg, deepening of anesthesia (Propofol concentrations 3.5 μg/ml), and administration of Nicardipine at 1–3 mg/h. Glycemia: varied between (0.98–1.21 g/l)

The tumor removal was followed by episodes of arterial hypotension (75 -45 mmHg), the conduct was to lighten the anesthesia, a volemic expansion with administration of norepinephrine at 2mg/h.

**Fig. 2:**
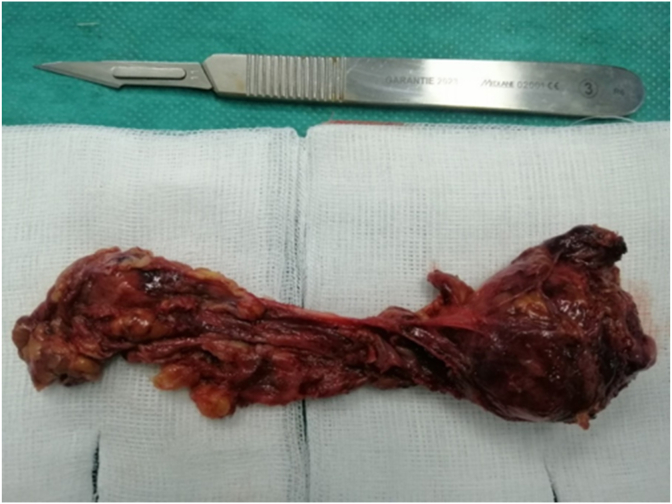
Macroscopic image of paraganglioma.

**Fig. 3:**
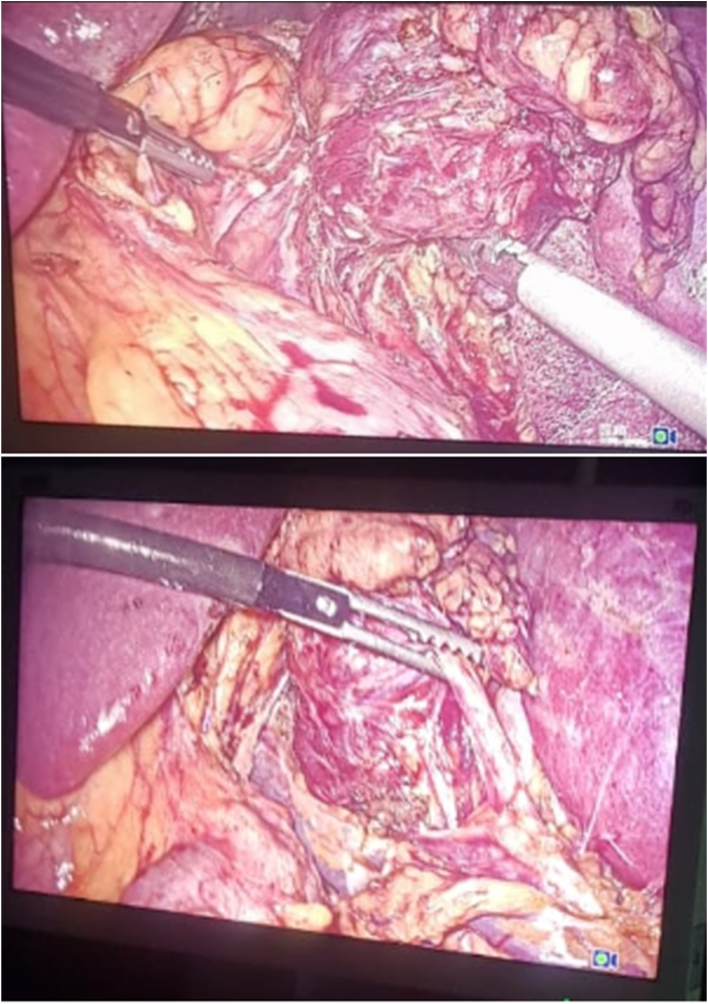
*Laparoscopic image of paraganglioma (A: posterior dissection of the mass, B: the* relations of the mass with the homolateral ureter).

For paraganglioma extraction, the procedure lasted 2 hours [Fig. 2, Fig. 3]., with an intraoperative diuresis of 500 ml and bleeding estimated at 80 cc.Norepinephrine infusion was gradually decreased and stopped just before extubation.

The patient was subsequently extubated with the following parameters BP: 140/75 mm hg HR: 90bpm, SaO2 100% and then sent to the intensive care unit for clinical and biological monitoring. Postoperative analgesia was started with paracetamol, nefopam and morphine. The antihypertensive treatment was resumed in front of the reappearance of hypertensive peaks.

**Fig. 4:**
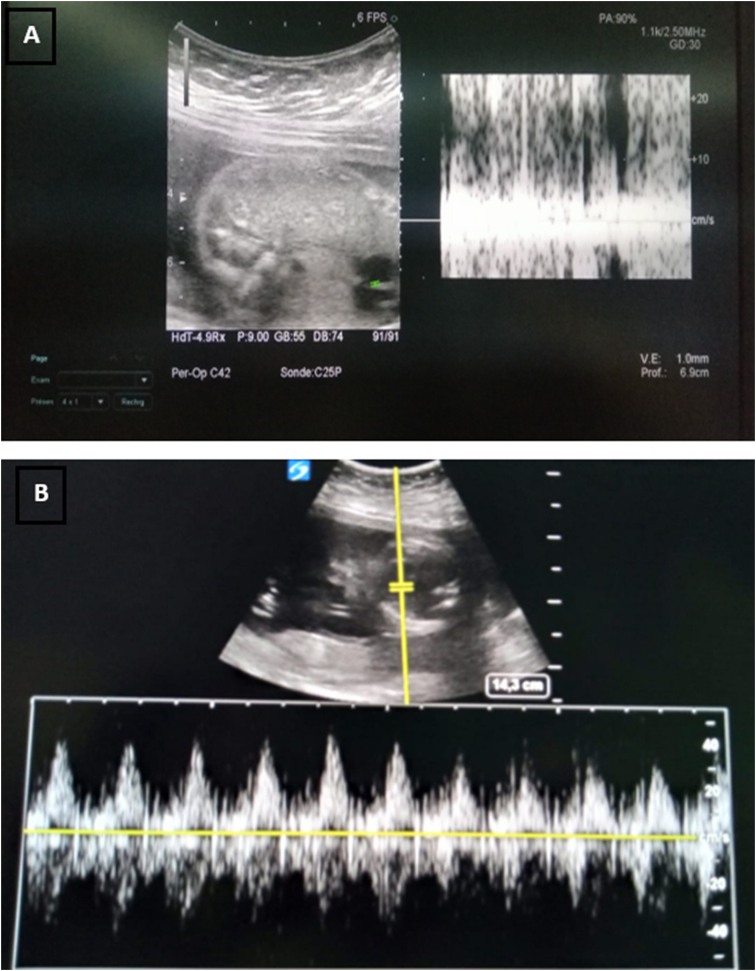
Obstetrical ultrasound showing the presence of fetal cardiac activity postoperatively (immediate: image A, and after 6 hours image B).

An obstetrical evaluation with a foeto-placental ultrasound immediately and 6 hours after the operation returned normal with the presence of fetal heart activity [Fig. 4].

2 days after the postoperative hospitalization in the intensive care unit, the patient was weaned from all drugs with good evolution and was transferred to the visceral surgery department for further management.

This cases report follows SCARE guidelines 2020 [5].

## 3 Discussion

The low incidence of endocrine tumors (pheochromocytoma or paraganglioma) in pregnancy (1/50,000 pregnancies) makes the description of this association rare in the literature [6,7]. This association is also underestimated because of the difficulty to distinguish pheochromocytomas and secretory paragangliomas responsible for secondary hypertension from other causes of gestational hypertension such as preeclampsia. The presence of other clinical signs: paroxysmal character, the triad: headache, sweat, palpitation, the presence or not of proteinuria can make the difference [8,9].

in the literature, authors have described the association between pheochromocytoma and paraganglioma (PPGL) in pregnancy; a recent study demonstrated that pheochromocytoma and paraganglioma (PPGL) in pregnancy is a rare entity and management of these patients is fraught with uncertainty [10]. This study, showed also that medical therapy should be initiated when paraganglioma is diagnosed in pregnancy, and antepartum surgery should be reserved for special circumstances [10].

We report in our study the perioperative management of a case of a parturient woman diagnosed with paraganglioma during her pregnancy after placing the patient on medical treatment and scheduling her for surgery at 25 weeks of amenorrhea.

On the maternal level, the signs are secondary to the increase in catecholamine secretion. They are clinically manifested by pallor, headaches, hyper sudation, thermophobia and tachycardia with hypertension which is often paroxysmal [11].

Glucose intolerance or even diabetes may be secondary to hypermetabolism linked to the action of catecholamines on glucido-lipid metabolism. Biological diagnosis is based on the determination of catecholamine derivatives in plasma and urine over 24 hours [12].Imaging during pregnancy is based essentially on abdominal ultrasound and/or abdomino-pelvic MRI and allows precise localization of the tumor [13].

On the fetal level, the placental passage of catecholamines is less important and limited to 10%, therefore the fetus is not too exposed to variations in secretion of these hormones, however, there is a risk of fetal hypoxia secondary to vasoconstriction of the utero-placental circulation [4,11].

Surgical excision remains the radical treatment, but the brutal disorder in catecholamines which it entails as well as the modified physiology of the pregnant woman imposes anesthetic implications with a particular and specific perioperative management.

In terms of mortality, there is no significant difference between pheochromocytoma and paraganglioma, it is 9% in the mother against 14% in the fetus [14].Therefore, Management requires more careful preoperative and postoperative resuscitation.

## 4 Conclusion

The anesthetic and surgical management of pheochromocytomas and paragangliomas in pregnant women presents a real challenge whose maternal and fetal morbi-mortality is non-negligible requiring a multidisciplinary management of different times: preoperative peri and postoperative. The prognosis is even worse if the diagnosis is not known. Otherwise, the mortality has largely decreased recently thanks to improved management.

## Consent

Written informed consent was obtained from the patient for publication of this case report and accompanying images. A copy of the written consent is available for review by the Editor-in-Chief of this journal on request.

## Ethical approval

The ethical committee approval was not required given the article type (case report).

## Sources of funding

This research did not receive any specific grant from funding agencies in the public, commercial, or not-for-profit sectors.

## Author contribution

AFTISS Fatima Zahra: study concept, Data collection; data analysis; writing review & editing.

EL MEZZIOUI Sanae: Study conception, data analysis.

EL AIDOUNI Ghizlane: Study conception, data analysis.

MERBOUH Manal: writing review & editing.

BERAJAA Sara: contributor.

NASRI Sihame: contributor.

BKIYAR Houssam: supervision and data validation.

BOUZIANE Mohamed: Contributor.

HOUSNI Brahim: supervision and data validation.

## Guarantor

AFTISS Fatima Zahra.

## Provenance and peer review

Not commissioned, externally peer-reviewed.

## Declaration of competing interest

They have no conflicts of interest for this report.
